# Rare earth elements in paddy fields from eroded granite hilly land in a southern China watershed

**DOI:** 10.1371/journal.pone.0222330

**Published:** 2019-09-11

**Authors:** Haibin Chen, Zhibiao Chen, Zhiqiang Chen, Qianyi Ma, Qingqing Zhang

**Affiliations:** 1 College of Geographical Sciences, Fujian Normal University, Fuzhou, Fujian, People’s Republic of China; 2 Key Laboratory of Humid Subtropical Eco-geographical Process (Fujian Normal University), Ministry of Education, Fuzhou, Fujian, People’s Republic of China; 3 School of History and Geography, Minnan Normal University, Zhangzhou, Fujian, People’s Republic of China; Zhongnan University of Economics and Law, CHINA

## Abstract

There are large amounts of ion-adsorption rare earth resources in the granite red soil region of southern China, and exploitation of rare earth elements (REEs) has caused serious soil erosion and soil pollution in the area. In this study, the spatial variability of soil REEs in Zhuxi watershed, Changting County, southern China, was analyzed using a geostatistics method. The analysis produced several important results: (1) The content of total rare earth elements (TREEs) in the soil samples ranged from 56.04 to 951.76 mg kg^−1^, with a mean value of 255.34 mg kg^−1^, which was higher than the background value of soil in China. The REE variables showed strong positive Ce anomalies and strong negative Eu anomalies, with mean values of 2.26 and 0.44, respectively. (2) The contents of TREEs in five subtypes of the soils were different, but they had broadly similar curves of chondrite-normalized REE patterns, with steeper patterns from La to Eu and flatter patterns from Eu to Y. (3) The spatial variability of light rare earth elements (LREEs) was mainly affected by natural factors, but the spatial variabilities of heavy rare earth elements (HREEs) and TREEs were influenced by the combination of natural factors and anthropogenic factors. Soil erosion can contribute significantly to REE migration, especially for HREEs. (4) The distribution of TREEs showed that the high content of TREEs was in the lowland of the western watershed. By comparing the distributions of TREEs in paddy fields and hilly land, we found that the area with a high content of TREEs was greater in paddy fields than in hilly land, so we deduced that REEs migrate from hilly land to the paddy field and accumulate in the soil there.

## Introduction

REEs as a group of 17 elements comprising 15 lanthanides (La, Ce, Pr, Nd,Pm, Sm, Eu, Gd, Tb, Dy, Ho, Er, Tm, Yb, and Lu) plus Y and Sc were defined by International Union for Pure and Applied Chemistry [1]. They have similar physical properties, chemical properties, and geochemical behaviors [2] and are typically divided into heavy rare earth elements (HREEs, Gd to Lu and Y) and light rare earth elements (LREEs, La to Eu) [3]. These elements are essential for a diverse and expanding array of high-technology applications, such as electric vehicles, energy-efficient lighting, and wind power [4].

China is the largest producer and exporter (> 97% of the world’s export volume) of these critical metals [2]. Southern China contains large amounts of granite weathering crust, which is enriched in ion-adsorption REEs in lateritic clay deposits [5]. These clay deposits have been exploited since the 1970s. The ion-adsorption REEs contained in these clay deposits are extracted easily, so under the impetus of profit, exploitation of the resource was weakly regulated for more than 30 years [6]. Ion-adsorption REEs constitute only 2.9% of China’s REE reserves, but these accounted for 35% of China’s REE production during 1988–2009 [7]. Due to weak regulation, the exploitation has left a legacy of environmentally damaging accidents and contamination [6].

Generally, low contents of REEs are presented in the soil, but REEs can accumulate in such environments because of the low mobility of these elements under anthropogenic influence [8,9]. The spatial distribution of REEs in the urban environment with regard to type of land use [10], and it has been found elevated environmental contamination in areas surrounding factories[11]. The exploitation activities are notorious for their adverse impacts on the environment [12]. The Zhuxi watershed, within which more than 50 illegal rare earth exploitation sites are scattered, was known for weak regulation of rare earth exploitation [13]. Our previous study had found that the agricultural soil environment in this area was moderately polluted by REEs, and it was treated for human health [14]. Identifying the spatial distribution characteristics of REEs is of great significance for the evaluation of soil pollution and the formulation of pollution prevention strategies [15]. However, the contamination characteristics of REEs in the watershed are not clear.

Geostatistics provides a set of statistical tools for incorporation of the spatial and temporal coordinates of observations in data processing, and its use has become popular in soil research since the 1980s [16]. Geostatistics has been utilized widely to quantify the spatial patterns of environmental variables [17]. Several attempts have been made to identify the variability of soil physical properties and heavy metals using a geostatistics method [17–20], but there has been little use for REEs, especially in the lateritic clay deposits of southern China.

The principal objectives of this study were to (1) obtain the REE contents in granite watershed and analyze their spatial variation and distribution, (2) investigate the REE pattern in different types of soil, (3) investigate the factors influencing REE distribution, and (4) investigate the differences of REE distribution between paddy fields and hilly land.

## Materials and methods

### Study area

**Fig 1:**
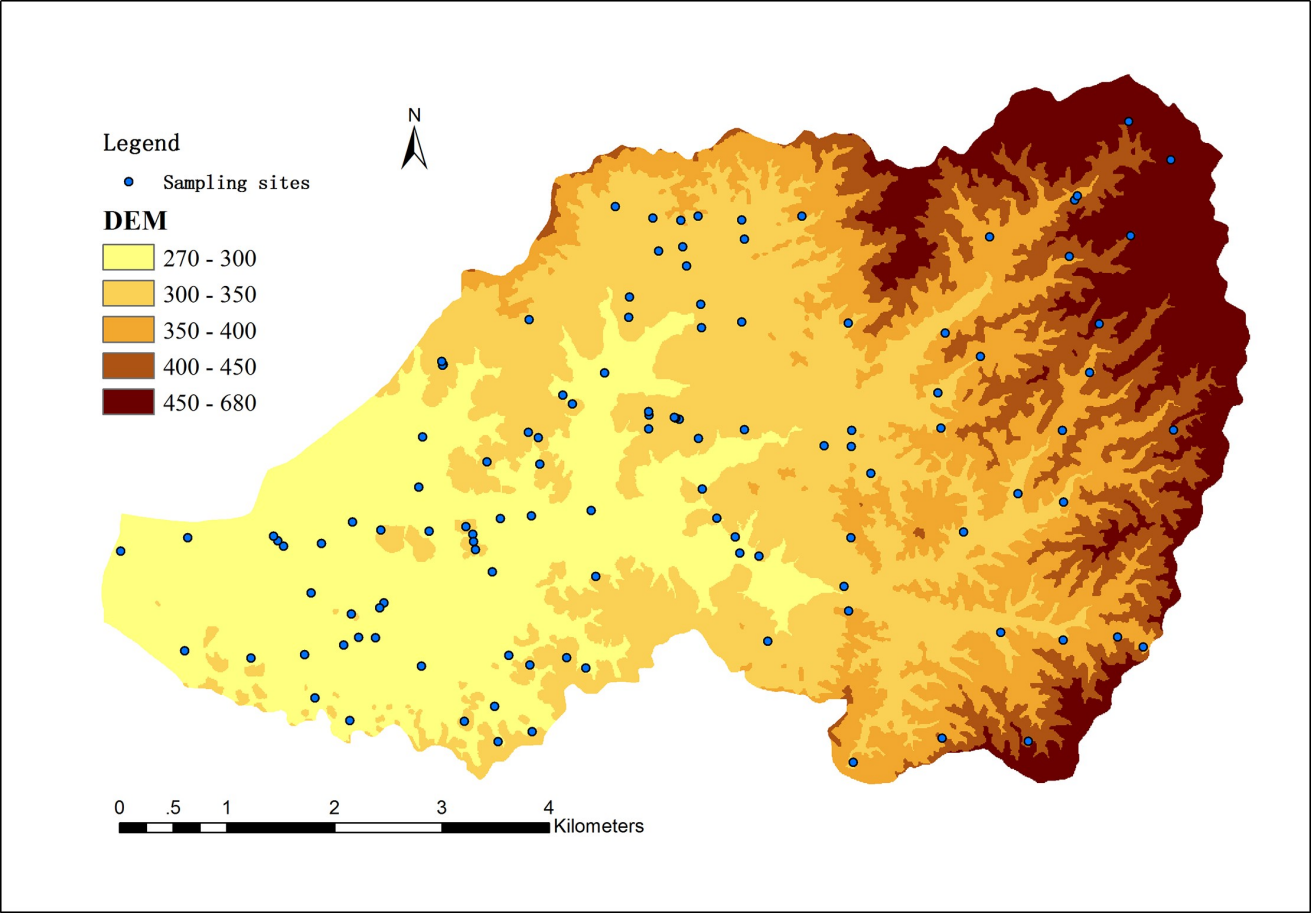
The distribution of soil sampling points and topography of the Zhuxi watershed.

The Zhuxi watershed (25°38'15''–25°42'55''N, 116°23'30''–116°30'30''E), with an area of 44.95 km^2^, is located at the center of Changting County, Fujian Province, southern China. It is known widely for its abundant rare earth mines as well as serious soil erosion. The landforms consist mainly of hills and low mountains. The topography is shown in Fig 1. This region is affected strongly by its warm and humid subtropical monsoon climate (the mean precipitation is 1,730.4 mm yr^-1^ and the mean annual temperature is 18.3°C), and the dominant soil type is red soil formed from coarse-grained biotitic granite [21]. Historically, the Zhuxi watershed had good vegetation cover, but due to weakly regulated exploitation and high-intensity interference from human activity, soil erosion has become a serious problem in this area.

### Sample collection

In total, 118 sampling sites were selected in the study area (Fig 1). The sampling sites were all located in the public area, just collecting soil samples, not involve any specials, that no specific permissions were required. And the field work was carried out with the assistance of the local Soil and Water Conservation Bureau. Among these, 52 sampling sites were located in a 1 km × 1 km grid node, and the other 66 sampling sites were selected according to the units generated by overlaying the maps of the land use layer, vegetation layer, soil group layer, and topography layer. This method ensured that small units were not neglected, which may be the case in simple random or systematic sampling methods [22]. At each sampling site, a mixed sample was collected from five sampling points of surface soil (0–20 cm). Roots were removed from the soil samples, the samples were then air-dried, and finally sieved through a series of sieves before analysis.

### Analytical methods

All soil samples were analyzed in the Key Laboratory of Humid Subtropical Eco-geographical Process (Fujian Normal University), Ministry of Education.

The soil samples for REE determination were crushed with an agate mill, and the ground samples were passed through a 0.149 mm polyethylene sieve. Soil samples of 0.1g were dissolved in a Milestone Microwave Laboratory System (Multiwave 3000, Anton Paar, Austria) in a combination solution of hydrofluoric, hydrochloric, and nitric acid (HF 40%: HCl 38%: HNO_3_ 70% = 1:1:3). All soil samples were analyzed for 15 REEs (Sc was excluded due to its different chemical properties and Pm is a trace material in nature) by inductively coupled plasma mass spectrometry (ICP-MS, X Series 2, Thermo Scientific, USA). The Detailed analytical procedures for the REE analysis are described by our previous paper [13].

### Data analysis

All statistical analyses were performed using SPSS19.0 software (SPSS Inc., Chicago, IL, USA). Normality was verified using the value of skewness and kurtosis. Natural logarithmic transformation was used to meet the assumptions of normality when the raw data did not obey a normal distribution, but the raw (untransformed) data are listed in the tables in this paper. From the analysis based on the data of the 15 REEs, we calculated six REE variables, total rare earth elements (TREEs), LREEs, HREEs, L/H, δCe, and δEu. TREEs is the sum of the 15 REEs; LREEs is the sum of La, Ce, Pr, Nd, Sm, and Eu; HREEs is the sum of Gd, Tb, Dy, Ho, Er, Tm, Yb, Lu, and Y; and L/H is the ratio of LREEs and HREEs. δCe and δEu are defined as follows:

$$\delta \mathrm{Ce}=\frac{{2\mathrm{Ce}}_{N}}{{\mathrm{La}}_{N}{+\mathrm{Pr}}_{N}}$$ (1)

$$\mathrm{\delta Eu}=\frac{{2\mathrm{Eu}}_{N}}{{\mathrm{Sm}}_{N}{+\mathrm{Gd}}_{N}}$$ (2)

where N refers to chondrite normalization [23]. The REE contents were normalized to chondrite for the purpose of comparison [24].

The geostatistical method was used to study the spatial variability of REEs in soils of the watershed. Geostatistics is based on the theory of a regionalized variable, which is distributed in space with spatial coordinates and shows spatial autocorrelation such that samples close together in space are more alike than those farther apart [25]. The geostatistics approach consists of the following two parts: the calculation of an experimental variogram from the data and model fitting, and estimation at unsampled locations [26,27]. The semivariogram of each soil property was constructed using the following model:

$$\gamma (h)=\frac{1}{2N(h)}{\sum_{i=1}^{N(h)}\left[Z(x_{i})-Z(x_{i}+h)\right]}^{2}$$ (3)

where γ (h) is the semi-variance for the internal distance class h, h is the lag interval, and N(h) is the total number of sample pairs for the lag interval h. Z(xi) is the measured sample value at point i, and Z(xi + h) is the measured sample value at point i + h. The semivariogram was calculated using GS+ 7.0 software (Gamma Design Software Inc., Plainwell, MI, USA) and the variability maps were created in ArcGIS 10.2 software (ESRI Inc., Redlands, CA, USA).

## Results and discussion

### REE contents and REE variables

The descriptive statistics of the REE contents and REE variables of the soils, which include mean, range, standard deviation, coefficient of variation, skewness, and kurtosis, are summarized in Table 1. All the data needed to obey a normal distribution in the subsequent semi-variance analysis, so the values of skewness and kurtosis were used to test the data for normal distribution. The value of skewness should be within the range of ±2 and the value of kurtosis should be less than 3; otherwise, it is regarded as an extreme [28]. The skewness and kurtosis results indicated that the REE contents and REE variables did not obey a normal distribution except for Ce and δEu. Therefore, we used logarithmic transformation to normalize the data. Thus, all the data successfully passed the test for normality.

**Table 1:** Statistical characteristics of REEs.

Variable	Min	Max	Mean	SD	CV (%)	Skewness	Kurtosis	Distribution type	Background value [31]
**La** **(mg kg**^**-1**^**)**	2.97	178.37	34.05	29.90	87.8	1.87/ -0.20*	7.47/ 2.6*	LN	39.7
**Ce** **(mg kg**^**-1**^**)**	23.16	181.91	93.53	37.70	40.38	0.21	2.28	N	68.4
**Pr** **(mg kg**^**-1**^**)**	0.66	76.63	9.04	10.23	113.18	3.48 / 0.01*	19.87/ 2.86*	LN	7.17
**Nd** **(mg kg**^**-1**^**)**	2.37	270.33	33.20	37.22	112.12	3.33/ -0.01*	18.32/ 2.8*	LN	26.40
**Sm** **(mg kg**^**-1**^**)**	0.62	57.02	7.38	8.22	111.36	2.96/ 0.13*	14.91/ 2.64*	LN	5.22
**Eu** **(mg kg**^**-1**^**)**	0.18	10.07	1.11	1.32	118.76	3.78 / 0.55*	22.04/ 3.01*	LN	1.03
**Gd** **(mg kg**^**-1**^**)**	1.26	59.10	8.41	8.41	100	2.90 / 0.38*	14.64/ 2.68*	LN	4.60
**Tb** **(mg kg**^**-1**^**)**	0.11	8.87	1.27	1.39	109.43	2.80 / 0.34*	12.95/ 2.57*	LN	0.63
**Dy** **(mg kg**^**-1**^**)**	0.78	48.51	7.92	8.30	104.78	2.64 / 0.34*	11.59/ 2.49*	LN	4.13
**Ho (mg kg**^**-1**^**)**	0.16	8.75	1.61	1.61	100	2.46 / 0.28*	10.41/ 2.44*	LN	0.87
**Er** **(mg kg**^**-1**^**)**	0.70	25.72	5.26	4.82	91.66	2.20 / 0.24*	8.87 / 2.29*	LN	2.54
**Tm** **(mg kg**^**-1**^**)**	0.10	3.81	0.76	0.70	90.14	2.13 / 0.19*	8.43 / 2.31*	LN	0.37
**Yb** **(mg kg**^**-1**^**)**	1.06	26.44	5.69	4.86	85.40	2.00 / 0.18*	7.75 / 2.26*	LN	2.44
**Lu** **(mg kg**^**-1**^**)**	0.15	3.69	0.83	0.70	83.82	1.91 / 0.13*	7.91 / 2.26*	LN	0.36
**Y** **(mg kg**^**-1**^**)**	4.81	157.23	45.28	38.86	85.81	1.13 / 0.07*	3.90 / 2.03*	LN	22.90
**LREEs (mg kg**^**-1**^**)**	38.11	627.95	178.3	101.32	56.82	1.35/-0.19*	5.70 / 2.71*	LN	147.92
**HREEs (mg kg**^**-1**^**)**	9.65	323.81	77.04	67.76	87.69	1.60 / 0.19*	5.30 / 2.13*	LN	38.84
**TREEs (mg kg**^**-1**^**)**	56.04	951.76	255.34	158.55	62.09	1.50/ 0.01*	5.84 / 2.52*	LN	186.76
**L/H**	0.44	10.73	3.28	1.93	58.94	1.39 /-0.3*	5.10 / 3.03*	LN	3.81
**δCe**	0.07	12.61	2.26	1.94	85.63	2.18/ -0.31*	6.58/ 1.37*	LN	0.91
**δEu**	0.12	0.71	0.44	0.109	24.60	-0.81	0.91	N	0.63

SD = standard deviation, CV = coefficient of variation

* the value after natural logarithm transformation

LN = Log normal, N = Normal

The coefficient of variation values (CVs) can be used to compare the discrete degree of a property. Low CVs correspond to a spatially homogeneous distribution, and high CVs correspond to a non-homogeneous distribution [29,30]. The CVs of Pr, Nd, Sm, Eu, Gd, Tb, Dy, and Ho were greater than 100%, which indicates a high variability. The CVs of La, Ce, Er, Tm, Yb, Lu, and Y were moderate, with fluctuation only from 40.38% to 91.66%. High CVs for REE contents have also been documented by other research [30]. Previous studies have shown that CVs of heavy metals originated from natural sources are relatively low, whereas CVs of heavy metals affected by anthropogenic activities are quite high [30–32]. It can be concluded that exploitation activities and soil erosion influence the differentiation of REEs greatly. The CVs of the 6 REE variables were moderate, with fluctuation from 24.6% to 87.69%. The CVs of LREEs were moderate compared to those of HREEs because LREEs were largely depended on the Ce, which had the lowest CV among the 15 REEs. This indicates that HREEs were affected more strongly by anthropogenic activities than LREEs.

The content of TREEs in the soil samples ranged from 56.04 to 951.76 mg kg^−1^, with a mean value of 255.34 mg kg^−1^. The mean value of TREEs is higher than the background value of soil in China (186.76 mg kg^-1^) [33] and higher than those in Japan (98 mg kg^−1^) [34] and Australia (105 mg kg^−1^) [35]. The content of LREEs ranged from 38.11 to 627.95 mg kg^−1^, with a mean value of 178.3 mg kg^−1^, and the content of HREEs ranged from 9.65 to 323.81 mg kg^−1^, with a mean value of 77.04 mg kg^−1^. The L/H value ranged from 0.44 to 10.73, with an average of 3.28, which shows that the content of LREEs is significantly higher than that of HREEs. This study also revealed strong positive Ce anomalies (2.26) and strong negative Eu anomalies (0.44), which is in agreement with the results of other studies in southern China, such as at Hainan Island [36] and in southern Jiangxi Province [37]. The contents of the 15 REEs tended to follow the Oddo–Harkins rule, which states that even atomic numbers are more frequent than their neighbors with odd atomic numbers. The order of the means of the 15 REE contents in the soil is Ce > Y > La > Nd > Pr > Gd > Dy > Sm > Yb > Er > Ho > Tb > Eu > Lu > Tm. Similar results were reported in other studies [14,30,34].

### REE patterns in different soil subtypes

**Fig 2:**
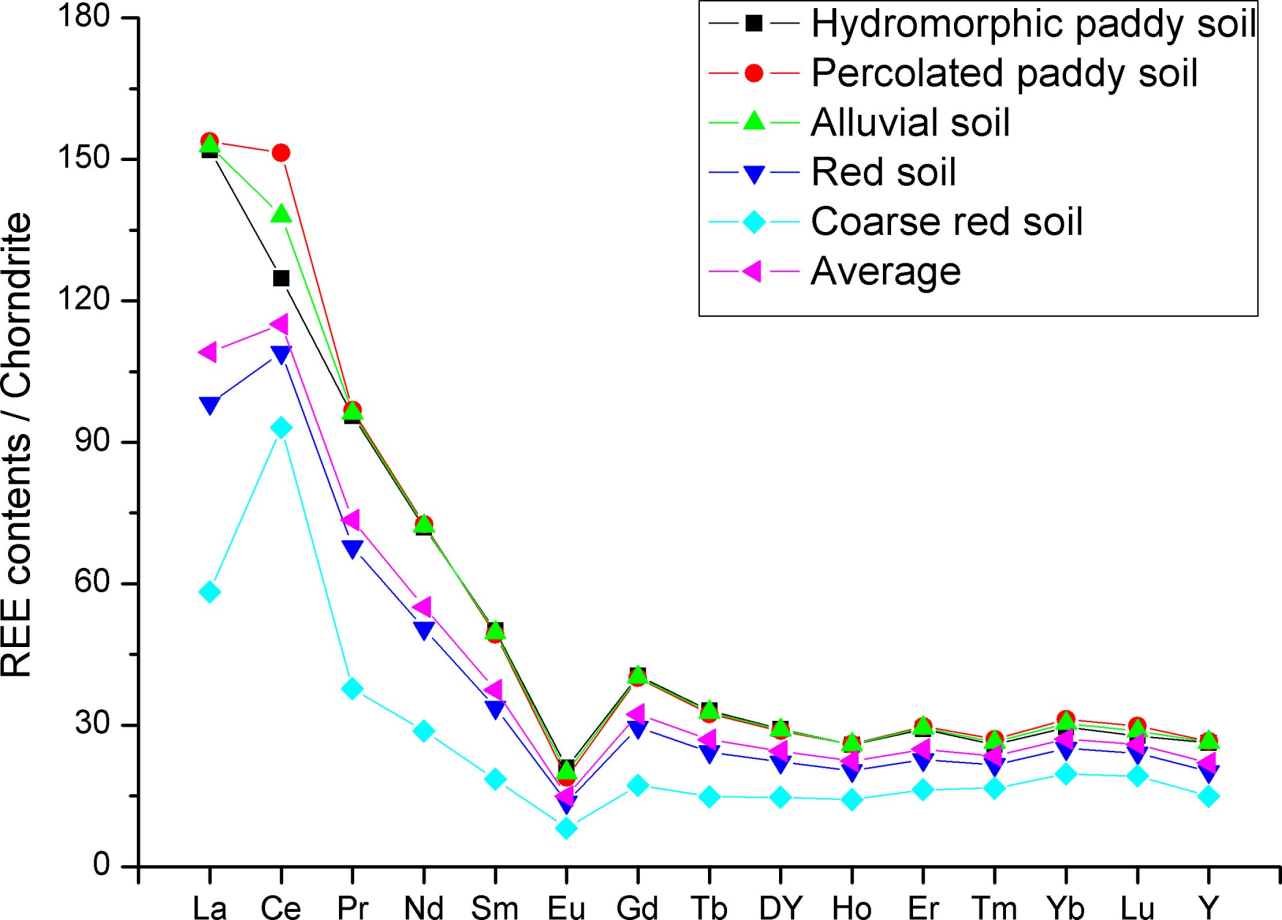
Chondrite-normalized REE patterns in different subgroups of soil.

The soil types in the study area include paddy soil, alluvial soil, and red soil. The area of red soil accounts for more than 74% of the total area. To distinguish the distribution of TREEs among the different soil types, the soil types were divided into subtypes. The subtypes of soil in the study area were hydromorphic paddy soil, percolated paddy soil, alluvial soil, red soil, and coarse red soil. The content of TREEs in the five soil subtypes is shown in Table 2. For comparison, the contents of TREEs were normalized to chondrite [24], as shown in Fig 2. The highest mean TREEs content of 330.36 mg kg^-1^ was determined in the percolated paddy soil, followed by the hydromorphic paddy soil, alluvial soil, red soil, and coarse red soil, respectively. Although there were some differences, the five subtypes of soil had similar curves of chondrite-normalized REE patterns, with steeper patterns from La to Eu and flatter patterns from Eu to Y.

**Table 2:** Statistical characteristics of TREEs in subgroups soils.

	Hydromorphic paddy soil (n = 8)	Percolated paddy soil (n = 12)	Alluvial soil (n = 3)	Red soil (n = 88)	Coarse red soil (n = 7)	Average (n = 118)
**La (mg kg**^**-1**^**)**	47.41	48.00	47.70	30.66	18.17	34.05
**Ce (mg kg**^**-1**^**)**	101.40	123.10	112.25	88.65	75.68	93.53
**Pr (mg kg**^**-1**^**)**	11.76	11.91	11.83	8.34	4.65	9.04
**Nd (mg kg**^**-1**^**)**	43.35	43.78	43.56	30.47	17.33	33.20
**Sm (mg kg**^**-1**^**)**	9.87	9.70	9.79	6.65	3.65	7.38
**Eu (mg kg**^**-1**^**)**	1.55	1.40	1.48	1.01	0.61	1.11
**Gd (mg kg**^**-1**^**)**	10.52	10.40	10.46	7.69	4.49	8.41
**Tb (mg kg**^**-1**^**)**	1.56	1.52	1.54	1.14	0.70	1.27
**DY (mg kg**^**-1**^**)**	9.43	9.31	9.37	7.16	4.74	7.92
**Ho (mg kg**^**-1**^**)**	1.86	1.87	1.87	1.47	1.03	1.61
**Er (mg kg**^**-1**^**)**	6.15	6.28	6.21	4.79	3.44	5.26
**Tm (mg kg**^**-1**^**)**	0.85	0.89	0.87	0.71	0.55	0.78
**Yb (mg kg**^**-1**^**)**	6.20	6.57	6.38	5.26	4.12	5.69
**Lu (mg kg**^**-1**^**)**	0.89	0.95	0.92	0.77	0.62	0.83
**Y (mg kg**^**-1**^**)**	54.15	54.70	54.42	41.49	30.83	45.28
**TREEs (mg kg**^**-1**^**)**	306.95	330.36	318.65	236.25	170.60	255.34

The content of a TREEs pattern is linked to the soil parent material [38,39], and the parent material in this study area is the same of coarse-grained granite, so all five soil subtypes showed similar patterns. Ample evidence has shown that REEs can be mobilized during weathering processes in the tropical and subtropical monsoon climates in southern China [23,40,41], which is why the REE content presents differences between the soil subtypes in this study. Our previous study determined that REEs migrate downhill under the influence of water flow and gravity [23]. The higher TREE contents of the hydromorphic paddy soil, percolated paddy soil, and alluvial soil than the red soil and coarse red soil are probably caused by differences of altitude. Comparing the TREE contents in the red soil and the coarse red soil, we found that the red soil only 19.09 mg kg^-1^ less than the average of all five subtypes of soil, and the coarse red soil was much lower than the average. Considering that the coarse red soil was located in the region where soil erosion has occurred seriously in the study area [21], we can conclude that soil erosion is one of the main factors for REE migration.

### Spatial variability of REEs

The REE contents in the soil may have been affected by intrinsic or extrinsic sources that cannot be discriminated by descriptive statistics. Thus, the spatial correlation structure of the REEs was explored by semivariogram. This method not only considered the randomness of the data, but also the spatial structure characteristics of the data. The best variogram model and parameters of REE contents and REE variables are listed in Table 3. They were fitted with spherical, exponential, linear, and Gaussian semivariograms with determination coefficient values (R^2^) ranging from 0.449 to 0.958. The R^2^ of all fitting results is greater than 0.3, indicates that the fitting results is better [42,43].

**Table 3:** Semivariogram models and interpolation parameters of REEs.

Variable	Model	Nugget(C_0_)	Sill(C_0_+C)	C_0_/(C_0_+C) (%)	Range (m)	R^2^
**La**	Spherical	0.064	0.796	8	750	0.499
**Ce**	Exponential	531	1463	36.3	1170	0.805
**Pr**	Spherical	0.072	0.902	8	780	0.531
**Nd**	Exponential	0.125	0.91	13.7	790	0.698
**Sm**	Exponential	0.114	0.891	12.8	1130	0.699
**Eu**	Spherical	0.045	0.697	6.5	510	0.451
**Gd**	Linear	0.545	0.673	80.1	5320	0.751
**Tb**	Exponential	0.481	0.802	60	3467	0.699
**Dy**	Linear	0.537	0.803	66.8	5201	0.863
**Ho**	Exponential	0.382	0.731	52.3	5300	0.717
**Er**	Exponential	0.395	0.655	60.3	5253	0.764
**Tm**	Exponential	0.265	0.664	39.9	5539	0.799
**Yb**	Spherical	0.304	0.612	49.7	5200	0.876
**Lu**	Linear	0.298	0.670	44.5	5320	0.958
**Y**	Exponential	0.468	0.722	64.6	5381	0.821
**LREEs**	Exponential	0.056	0.318	17.5	1275	0.449
**HREEs**	Exponential	0.328	0.716	45.8	5586	0.801
**TREEs**	Exponential	0.226	0.336	67.4	3571	0.579
**L/H**	Exponential	0.156	0.368	42.3	4710	0.765
**δCe**	Linear	0.595	0.682	87.3	5118	0.521
**δEu**	Gaussian	0.006	0.012	47.4	2061	0.713

The ratio of nugget effect (C_0_) over sill (C_0_ + C) is the nugget variance, which expresses the percent of total semi-variance [30]. It was used to judge the spatial dependency of the REE parameters and provide a quantitative basis for interpolating unsampled locations. If the ratio was equal to or lower than 25%, the variables were considered strongly dependent; if it was between 25% and 75%, the variables were considered moderately dependent; and if it was greater than 75%, the variables were considered weakly dependent [44,45]. In this study, the semivariograms indicated strong spatial dependence for REEs such as La, Pr, Nu, Sm, Eu, and LREEs but indicated moderate spatial dependence for Ce, Tb, Dy, Ho, Er, Dy, Yb, Lu, Y, HREEs, TREEs, L/H, and δEu. Only Gd and δCe showed weak spatial dependence. The strong spatial dependence of REEs in the soil may be the result of natural factors, such as strong pedogenesis [39]. Thus, we can see that the 6 LREEs except for Ce, present strong spatial dependence. With the variables of LREEs presenting strong spatial dependence, we concluded that the LREEs in the soil were affected mainly by natural factors. Moderate spatial dependence indicates that anthropogenic factors changed the soil texture spatial correlation through activities such as farming, management, practices, industrial production, and other human activities [46,47]. The 9 HREEs, except for Gd, presented moderate spatial dependence, which indicates that HREEs were more affected by anthropogenic factors than LREEs. The TREEs were influenced by a combination of natural and anthropogenic factors [48]. In the case of Gd, as the transition element between LREEs to HREEs, the difference between sampling sites may have weakened the spatial correlation. δCe also presented weak spatial dependence caused by a complicated oxidation-deoxidation environment.

The range values showed large variability among the REEs, which can be a useful principle for mapping [49,50]. The results indicate that the spatial correlations (range) of the REE contents and REE variables vary widely from 510 m (Eu) to 5586 m (HREEs). The different ranges of the spatial dependence among the REEs may be a result of the parent material, erosion–deposition factors, or topography [48]. A large range indicates that the observed values are affected over greater distance by other values of the parameter compared to the parameters with smaller ranges [51]. Thus, the range of 5586 m for HREEs implies that HREEs values influenced neighboring values over greater distances than did values of other soil variables.

### Spatial distribution of REEs

**Fig 3:**
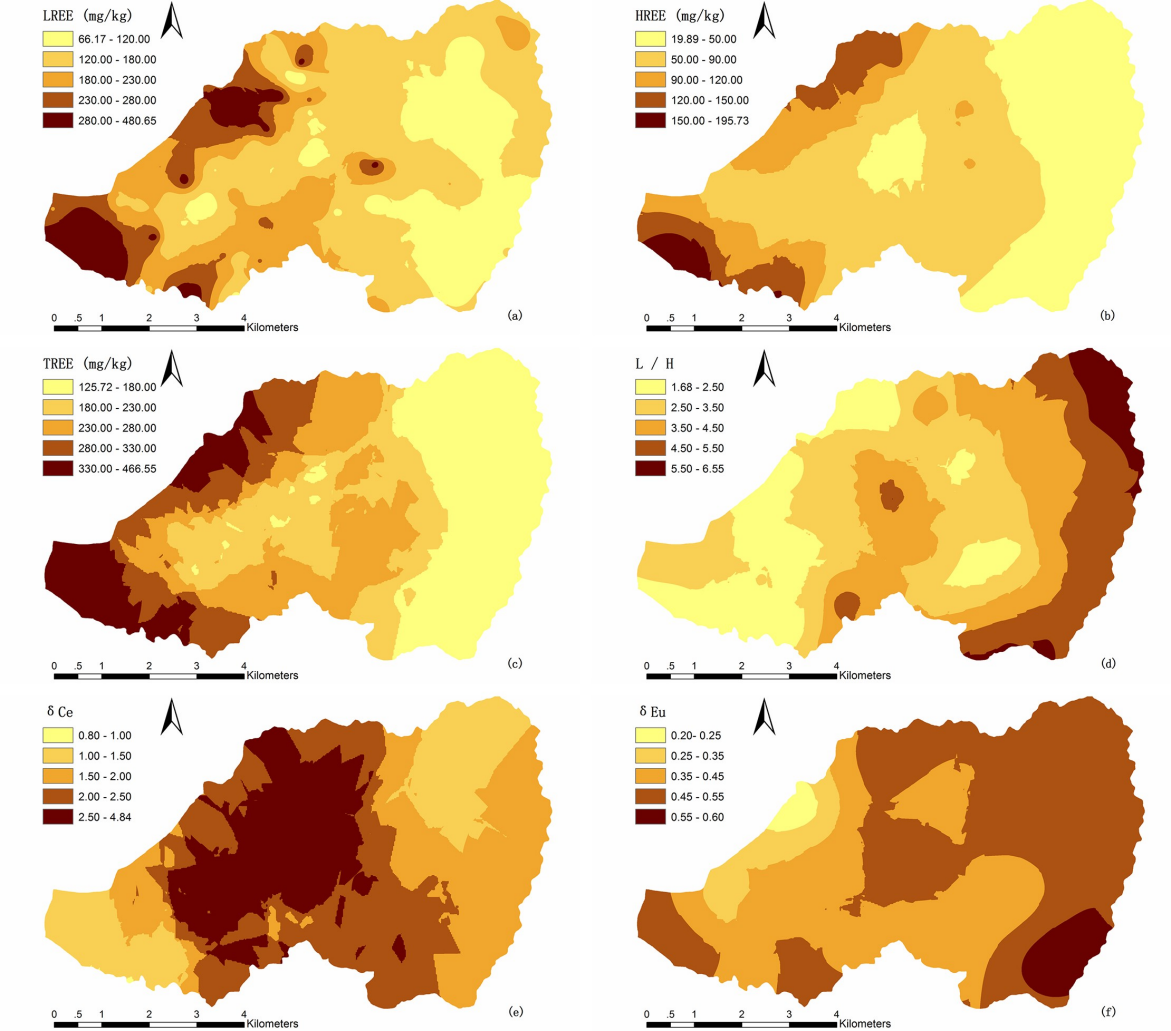
Spatial distribution maps of REE variables: A LREE, b HREE, c TREE, d L/H, e δCe, f δEu.

To express the REE distribution clearly, six REE variables were chosen for spatial interpolation by Kriging. The spatial distributions of these REE variables are shown in Fig 3. It is useful to identify areas with high contents of REEs and assess the possible variation of REEs in the watershed.

The distribution map of LREEs presents an irregular trend in different directions and some discontinuous high content areas located in the central part of the watershed. On the whole, areas with a high content of LREEs are located in the west and northwest. The map of HREEs distribution shows an increasing trend from east to west and northwest, except for the small plot of low content in the central part of the watershed. The distribution map of TREEs presents a trend similar to the distribution trend of HREEs. The topography of the watershed is tilted from east to west, and a previous study found that REEs can migrate downhill to lower sites by water flow and gravity [23], so the high REE content area is located in the lowland of the west. The central part of the watershed is the area where soil erosion was serious [21]. The distribution map of L/H shows a decreasing trend from east to west, except for the central part of the watershed. The soil erosion caused migration of REEs and preferential release of HREEs resulting in a greater depletion of HREE than LREE [52,53]. Thus, we further demonstrated that soil erosion caused migration of HREEs more than LREEs.

The depletion or enrichment of Ce and Eu usually occurs in nature due to their oxidation state and mobility under different oxidation–reduction conditions [54]. The distribution of δCe shows a decreasing trend from the central part of the area to the east and to the west. A strongly positive Ce anomaly area (δCe >2.5) is located in the north-central part of the watershed, and it may be influenced by weathering processes [4] and soil erosion. Ce^3+^ oxidized to Ce^4+^ under the exogenous environment was stored in the surface soil stably, and soil erosion caused losses of other REEs producing positive Ce anomaly development in this area [54]. The distribution of δEu shows a decreasing trend from southeast to northwest. The Eu anomaly is affected mainly by parent material, and the development process of the soil is also a process of Eu loss increase [30]. Thus, because the parent material originates form coarse-grained biotitic granite, the variation of negative Eu anomaly was small, ranging from 0.20 to 0.60. The strongly negative Eu anomaly area is located in the northwestern part of the watershed where the degree of soil maturation is high. The Ce positive anomaly and Eu negative anomaly indicate that differentiation occurred between Ce, Eu, and other REEs in the weathering process of the parent rock [30,55].

### Distribution of TREEs between paddy fields and hilly land

From the analysis above, we concluded that REEs migrated due to soil erosion. To verify whether REEs accumulated in lowland, where paddy fields are largely located, we compared the TREE distribution between paddy fields and hilly land. The areas of different classes of TREEs are shown in Table 4.

**Table 4:** Distribution ratio of TREEs between paddy fields and hilly land.

TREE (mg kg^-1^)		<180	180–230	230–280	280–330	>330	Total
**Hilly land**	Area (km^2^)	14.06	6.89	7.14	3.33	2.76	34.18
	Percentage (%)	41.14	20.16	20.89	9.74	8.07	100
**Paddy fields**	Area (km^2^)	0.65	2.92	3.09	2.08	2.03	10.77
	Percentage (%)	6.05	27.12	28.66	19.31	18.86	100

The Table 4 shows that the area of TREE content lower than 180 mg kg^-1^ (background value of soil in China is 187.60 mg kg^-1^) [33] accounted for more than 41.14% of the distribution in the hilly land but for only 6.05% in the paddy fields. Additionally, in the class of 180–230 mg kg^-1^ (background value of soil in Fujian Province is 223.47 mg kg^-1^) [13], the area in the hilly land accounted for 20.16%, which means that about 61.30% of the area of TREE content in the hilly land is lower than the Fujian background value. However, the area of TREE content in paddy fields higher than the Fujian background value accounted for more than 66.83% of the total area. The percentage of high content classes of TREEs was greater in paddy fields than in hilly land. The parent material originated from the coarse-grained biotitic granite in the watershed, so the distribution of REEs in soil derived from the parent material should be similar in different regions. The difference of REEs in soil between hilly land and paddy fields further indicates that REEs migrate in the watershed. Combining these results with the previous findings, we suggest that soil erosion can significantly contribute to the migration of REEs in the soil. The REEs migrated from hilly land to the paddy fields in the watershed and then accumulated there.

## Summary and conclusions

The content of REEs in the soil of the granite area in southern China was higher than the average value in China and higher than the average value in Fujian Province. The trend of REEs in soil followed the decreasing order of Ce > Y > La > Nd > Pr >Gd > Dy > Sm > Yb > Er > Ho > Tb > Eu > Lu > Tm and obeyed to the Oddo–Harkins rule, presenting strong positive Ce anomalies and strong negative Eu anomalies, which is similar to the results of other studies in southern China. Although the contents of TREEs in the five subtypes soil were different, they had broadly similar curves of chondrite-normalized REE patterns, indicating that the REE pattern was affected by parent material.

The nugget variance indicated that the spatial variability of LREEs was mainly affected by natural factors, while the spatial variabilities of HREEs and TREEs were influenced by the combination of natural factors and anthropogenic factors. Soil erosion can significantly contribute to REE migration, especially for HREEs. The distribution of TREEs showed that the high-TREE content area was located in the lowland of the western watershed. Additionally, the percentages of high content classes of TREEs were greater in paddy fields than in hilly land, so we conclude that REEs can migrate from hilly land to the paddy fields in the watershed.

## Supporting information

S1 File Dataset. (ZIP)

## References

[1] IUPAC. Nomenclature of Inorganic Chemistry: IUPAC Recommendations 2005 Cambridge University Press, Cambridge, UK 2005.

[2] Sukumaran PV. Elements that rule the world: Impending REE metal crisis. J Geo Soc India. 2012;80(2):295–295. 10.1007/s12594-012-0144-6

[3] Liu J, Jiang Y, Xie P, Li Q. Geochemistry of rare earth elements and yttrium in a Ge-poor coal from the Wulantuga ore deposit, Inner Mongolia, North China. Int J Coal Sci Technol. 2014;1(4):390–394. 10.1007/s40789-015-0052-7

[4] Long KR, Gosen BSV, Foley NK, Cordier D. The Principal Rare Earth Elements Deposits of the United States: A Summary of Domestic Deposits and a Global Perspective. Non-Renewable Resource Issues. Springer, Netherlands. 2010;131–155. 10.1007/978-90-481-8679-2_7

[5] Gao Z, Zhou Q. Contamination from rare earth ore strip mining and its impacts on resources and eco-environment. Chinese J Eco. 2011;12(12):2915–2922.

[6] Kynicky J, Smith MP, Xu C. Diversity of rare earth deposits: The key example of China. Elements. 2012;8(5):361–367. 10.2113/gselements.8.5.361

[7] Su W. Economic and Policy Analysis of China's Rare Earth Industry Beijing: China Financial and Economic Publishing House 2009.

[8] Cao X, Wang X, Zhao G. Assessment of the bioavailability of rare earth elements in soils by chemical fractionation and multiple regression analysis. Chemosphere. 2000;40(1):23–28. 10.1016/S0045-6535(99)00225-8 10665441

[9] D'Aquino L, Morgana M, Carboni MA, Staiano M, Antisari MV. Re M, et al Effect of some rare earth elements on the growth and lanthanide accumulation in different Trichoderma strains. Soil Biol Biochem. 2009;41(12):2406–2413. 10.1016/j.soilbio.2009.08.012

[10] Klaudia B, Marta L, Jolanta K, Anna B, Mirosław M, Przemysław N, et al Relations between rare earth elements accumulation in Taraxacum officinale L. and land use in an urban area—A preliminary study. Ecol Indic. 2018;94(1):22–27. 10.1016/j.ecolind.2018.06.046

[11] Wang B, Xia D, Yu Y, Chen H, Jia J. Source apportionment of soil-contamination in Baotou City (North China) based on a combined magnetic and geochemical approach. Sci Total Enviro. 2018;642:95–104. 10.1016/j.scitotenv.2018.06.050 29894886

[12] Wang L, Guo Z, Xiao X, Chen T, Liao X, Song J, et al Heavy metal pollution of soils and vegetables in the midstream and downstream of the Xiangjiang River, Hunan Province. J Geog Sci. 2008;18(3):353–362. 10.1007/s11442-008-0353-5

[13] Li X, Chen Z, Chen Z. Distribution and fractionation of rare earth elements in soil-water system and human blood and hair from a mining area in southwest Fujian Province, China. Environ Earth Sci. 2014;72(9):3599–3608. 10.1007/s12665-014-3271-0

[14] Li X, Chen Z, Chen Z, Zhang Y. A human health risk assessment of rare earth elements in soil and vegetables from a mining area in Fujian Province, Southeast China. Chemosphere. 2013;93(6):1240–1246. 10.1016/j.chemosphere.2013.06.085 23891580

[15] Li F, Zhang J, Liu W, Liu J, Huang J, Zeng G. An exploration of an integrated stochastic-fuzzy pollution assessment for heavy metals in urban topsoil based on metal enrichment and bioaccessibility. Sci Total Enviro. 2018,644:649–660. 10.1016/j.scitotenv.2018.06.366 29990913

[16] Webster R, Burgess TM. Optimal interpolation and isarithmic mapping of soil properties IIIchanging drift and universal Kriging. Europ J Soil Sci. 1980;31(3):505–524. 10.1111/j.1365-2389.1980.tb02085.x

[17] Webster R, Oliver MA. Geostatistics for environmental scientists. New York: John Wiley & Sons 2001.

[18] Hu W, Shao M, Wan L, Si B. Spatial variability of soil electrical conductivity in a small watershed on the Loess Plateau of China. Geoderma. 2014;230-231(230):212–220. 10.1016/j.geoderma.2014.04.014

[19] Wang Y, Shao M, Gao L. Spatial variability of soil particle size distribution and fractal features in water-wind erosion crisscross region on the Loess Plateau of China. Soil Sci. 2010;175(12):579–585. 10.1097/SS.0b013e3181fda413

[20] Barik K, Aksakal EL, Islam KR, Sari S, Angin I. Spatial variability in soil compaction properties associated with field traffic operations. Catena. 2014;120(3):122–133. 10.1016/j.catena.2014.04.013

[21] Bai L, Chen Z, Chen Z. Soil Fertility Self-development Under Ecological Restoration in the Zhuxi Watershed in the Red Soil Hilly Region of China. J Mt Sci. 2014;11(5):1231–1241. 10.1007/s11629-014-3056-7

[22] Grinand C, Arrouays D, Laroche B, Martin MP. Extrapolating regional soil landscapes from an existing soil map: Sampling intensity, validation procedures, and integration of spatial context. Geoderma. 2008;143(1):180–190. 10.1016/j.geoderma.2007.11.004

[23] Chen Z, Chen Z, Bai L. Rare earth element migration in gullies with different Dicranopteris dichotoma, covers in the Huangnikeng gully group, Changting County, Southeast China. Chemosphere. 2016;164:443–450. 10.1016/j.chemosphere.2016.08.123 27599011

[24] Masuda A, Nakamura N, Tanaka T. Fine structures of mutually normalized rare-earth patterns of chondrites. Geochim Et Cosmochim Acta. 1973;37(2):239–248. 10.1016/0016-7037(73)90131-2

[25] Black K, Creamer RE, Xenakis G, Cook S. Improving forest soil carbon models using spatial data and geostatistical approaches. Geoderma. 2014;232-234(12):487–499. 10.1016/j.geoderma.2014.05.022

[26] Jang C, Chen S, Kuo Y. Applying indicator-based geostatistical approaches to determine potential zones of groundwater recharge based on borehole data. Catena. 2013;101(3):178–187. 10.1016/j.catena.2012.09.003

[27] Wang J, Yang R, Feng Y. Spatial variability of reconstructed soil properties and the optimization of sampling number for reclaimed land monitoring in an opencast coal mine. Arab J Geosci. 2017;10(2):46 10.1007/s12517-017-2836-0

[28] Reimann RC, Filzmoser P, Garrett RG, Dutter R. Statistical Data Analysis Explained: Applied Environmental Statistics with R Wiley 2008.

[29] Karanlik S, Aqca N, Yalcin M. Spatial distribution of heavy metals content in soils of Amik Plain (Hatay, Turkey). Environ Monit Assess.2011;173(1–4):181–191. 10.1007/s10661-010-1380-0 20221796

[30] Wang L, Liang T. Geochemical fractions of rare earth elements in soil around a mine tailing in Baotou, China. Sci Rep. 2015;5(5):12483 10.1038/srep12483 26198417 PMC4510494

[31] Baran A, Wieczorek J. Application of geochemical and ecotoxicity indices for assessment of heavy metals content in soils. Arch Environ Prot. 2015;41(2):54–63. 10.1515/aep-2015-0019

[32] Delavar MA, Safari Y. Spatial distribution of heavy metals in soils and plants in Zinc Town, northwest Iran. Int J Enviro Sci Technol. 2016;13(1):1–10. 10.1007/s13762-015-0868-0

[33] Wang L, Zhang S, Gao X, Liu S, Wang Y, Sun J, et al Geochemical characteristics of Rare Earth Elements in different types of soils in China. J Rare Earths. 1998;(1):52–59.

[34] Taylor SR, Mclennan SM. The geochemical evolution of the continental crust. Rev Geophy. 1995;33(2):241–265. 10.1029/95RG00262

[35] Diatloff E, Asher CJ, Smith FW. Concentrations of rare earth elements in some Australian soils. Aust J Soil Res. 1996;34(5):735–747. 10.1071/SR9960735

[36] Wang P, Zhao Z, Wang J, Zhang Z, Lu S. Spatial distribution of REE elements contents in arid area of southwest Hainan Island. J Arid Land Resour Environ. 2012;26(5):83–87.

[37] Gao X, Zhang S, Wang L, Wang Y. REE and its relation with the mineral fraction in a typical geographic landscape in Ganxian, Southern Jiangxi. Geogr Res. 1999;18(3):241–246.

[38] Compton JS, White RA, Smith M. Rare earth element behavior in soils and salt pan sediments of a semi-arid granitic terrain in the Western Cape, South Africa. Chem Geol. 2003;201(3):239–255. 10.1016/S0009-2541(03)00239-0

[39] Davranche M, Grybos M, Gruau G, Pedrot M, Dia A, Marsac R. Rare earth element patterns: A tool for identifying trace metal sources during wetland soil reduction. Chem Geol. 2011;284(1):127–137. 10.1016/j.chemgeo.2011.02.014

[40] Gao X, Shen Z, Wang L. Environmental chemistry of rare earth elements (REEs) in the cultivated soil of a typical REE mine in the southern Jiangxi. Soil Enviro Sci. 2001;10:11–13.

[41] Tang N, Li J, Li Q. The distribution of rare earth elements sum and component of lateritic red soil and red soil in Fujian Province. Chin J Soil Sci. 1993;24(5):207–210.

[42] Al-Omran AM, Aly AA, Al-Wabe M I, Al-Shayaa MS, Sallam AS, Nadeem M E. Geostatistical methods in evaluating spatial variability of groundwater quality in al-kharj region, saudi arabia. Appl Water Sci. 2017;7(7):4013–4023. 10.1007/s13201-017-0552-2

[43] Seyedmohammadi J, Esmaeelnejad L, Ramezanpour H. Geospatial modelling for optimum management of fertilizer application and environment protection. Model Earth Syst Environ, 2017;3(1),28 10.1007/s40808-017-0296-x

[44] Cambardella CA, Moorman TB, Novak JM, Parkin TB, Karlen DL, Turco RF, et al Field-scale variability of soil properties in central Iowa soils. Soil Sci Soc Am J. 1994;58(5):1501–1511. 10.2136/sssaj1994.03615995005800050033x

[45] Xie Y, Li X, Wang J, Christakos G, Hu M, An L, et al Spatial estimation of antibiotic residues in surface soils in a typical intensive vegetable cultivation area in China. Sci Total Enviro. 2012;430(430):126–131. 10.1016/j.scitotenv.2012.04.071 22634559

[46] Fiket Ž, Medunić G, Kniewald G. Rare earth elements distribution in soil nearby thermal power plant. Enviro Earth Sci. 2015; 75(7): 1–9. 10.1007/s12665-016-5410-2

[47] Brito P, Prego R, Mil-homens M, Caçador I, Caetano M. Sources and distribution of yttrium and rare earth elements in surface sediments from Tagus estuary, Portugal. Sci Total Enviro. 2018; 621: 317–325. 10.1016/j.scitotenv.2017.11.245 29190555

[48] Li K, Liang T, Wang L, Yang Z. Contamination and health risk assessment of heavy metals in road dust in Bayan Obo Mining Region in Inner Mongolia, North China. J Geogr Sci. 2015; 25(12): 1439–1451. 10.1007/s11442-015-1244-1

[49] Utset A, Ruiz M, Herrera J, Leonc DPD. A geostatistical method for soil salinity sample site spacing. Geoderma. 1998; 86(1–2): 143–151. 10.1016/S0016-7061(98)00037-8

[50] Fu W, Tunney H, Zhang C. Spatial variation of soil nutrients in a dairy farm and its implications for site-specific fertilizer application. Soil Till Res, 2010;106(2):185–193. 10.1016/j.still.2009.12.001

[51] Lopez-Granados F, Jurado-Exposito M, Atenciano S, Garcia-Ferrer A, Orden MSDL, Garcia-Torres L. Spatial variability of agricultural soil parameters in southern Spain. Plant & Soil. 2002;246(1):97–105. 10.1023/A:1021568415380

[52] Aubert D, Probst A, Stille P. Distribution and origin of major and trace elements (particularly REE, U and Th) into labile and residual phases in an acid soil profile (Vosges Mountains, France). Appl Geochem. 2004;19(6):899–916. 10.1016/j.apgeochem.2003.11.005

[53] Migaszewski ZM, Gałuszka A, Dołe˛gowska S, Hałas S, Krzciuk K, Gebus B. Assessing the impact of Serwis mine tailings site on farmers’ wells using element and isotope signatures (Holy Cross Mountains, south-central Poland). Environ Earth Sci. 2015;74(1):629–647. 10.1007/s12665-015-4067-6

[54] Semhi K, Chaudhuri S, Clauer N. Fractionation of rare-earth elements in plants during experimental growth in varied clay substrates. Appl Geochem. 2009;24(3):447–453. 10.1016/j.apgeochem.2008.12.029

[55] Tyler G. Rare earth elements in soil and plant systems—A review. Plant & Soil. 2004;267(1/2):191–206. 10.1007/s11104-005-4888-2

